# Process Model for Transitioning Care Responsibility to Adolescents and Young Adults with Biliary Atresia: A Secondary and Integrative Analysis

**DOI:** 10.3390/nursrep15080308

**Published:** 2025-08-21

**Authors:** Katsuhiro Hiratsuka, Nobue Nakamura

**Affiliations:** 1 Department of Nursing, School of Nursing and Rehabilitation Sciences, Showa Medical University, 1865, Tokaichibacho, Yokohama Midori-ku, Yokohama 226-8555, Japan; 2 Department of Nursing Science, Uekusa Gakuen University, 1639-3, Oguracho, Chiba Wakaba-ku, Chiba 264-0007, Japan; nobu-nakamura@uekusa.ac.jp

**Keywords:** biliary atresia, transition of care, family-centered nursing, self-management, adolescent health, process model

## Abstract

**Background/Objectives:** This study conducted a secondary and integrative analysis of qualitative data on adolescents and young adults (AYAs) with biliary atresia who survive with their native livers. These individuals struggle with independence and self-care due to prolonged parental involvement. Prior studies have insufficiently clarified how AYAs and parents jointly navigate daily responsibility transitions during this period. Therefore, we aimed to elucidate this process and develop a practical model to support nursing care. **Methods:** Semi-structured interview data from eight adolescent–parent dyads (one male and seven females, aged 17–25; one father and seven mothers, aged 40–60) were reanalyzed using the modified grounded theory approach. By reframing the analytical focus on dyadic interactions, four transition phases were identified, which were then integrated with the findings of two prior studies to construct an integrative process model. **Results:** The transition comprised four phases: (1) parent-led recuperation, (2) a vicious cycle of control and rebellion, (3) passing the axis of responsibility, and (4) aligning the parent–child rhythm to create a patient-centered life. The transition processes were shaped by changes in cognition and behavior. The model illustrates mutual adaptation through communication, negotiation, and reflection, identifying opportunities for nursing intervention. **Conclusions:** This process model offers a practical framework for nurses to assess readiness for care transitions, support transitional role shifts, and co-develop care strategies. The model provides insights into relationship-based communication and shared decision-making in transitional care by capturing the relational dynamics between AYAs and their parents.

## 1. Introduction

### 1.1. Background

Advancements in medical care have improved survival for children with chronic illnesses, highlighting the critical need for support during their transition to adulthood [1,2]. For adolescents and young adults (AYAs) with childhood-onset conditions, fostering independence and self-care is essential. Various studies highlight the vulnerability of these AYAs, including physical, social, and emotional health issues, as well as the risk-taking behaviors commonly observed during this developmental period [2], such as substance use, physical inactivity, risky sexual activity, and medication non-adherence, which may further complicate their health status and care transition [3]. These challenges are shaped by early relationships with parents and clinicians, including overprotection and surrogate decision-making [4]. International guidelines generally recommend a developmentally appropriate, individualized approach to the transition process rather than a rigid age-based framework. However, some guidelines provide suggested age benchmarks for adolescents who do not have (or do not require) formal education, health, or social care plans. For instance, the UK’s NICE (NG43) and the U.S.-based “Got Transition” suggest initiating planning by early adolescence (ages 12–14) and emphasize gradual responsibility transfer aligned with individual readiness and shared decision-making [5,6].

Biliary atresia, a progressive fibroinflammatory disease of the biliary system, is a leading cause of neonatal cholestasis. Initial treatment involves the Kasai portoenterostomy, but 60–75% of patients still require liver transplantation by age 18 [7]. Thus, AYAs who survive with their native livers continue to face uncertainty regarding future transplantation—a situation that affects both their quality of life and capacity for self-management [8].

In Japan, 95% of living-donor liver transplants (LDLTs) are from parents [9]. This creates a unique emotional context: parents often hesitate to transfer responsibility, torn between nurturing their child and confronting the possibility of becoming a living donor [10]. As a result, support for the transition process must also consider the parents’ psychological states. Compared to other populations with chronic illnesses, AYAs with biliary atresia and their parents face greater emotional strain, with studies reporting lower mental health-related quality of life among parents involved in transplant-related decision-making, especially when they perceive their child’s condition as poor [11].

### 1.2. The Aim of the Present Study

Given the emotionally complex and relational nature of responsibility transition in biliary atresia, this study adopted a dyadic lens to explore how adolescents and young adults (AYAs) and their parents jointly navigated the transfer of daily responsibilities. We aimed to develop a process model that captures the intersubjective dynamics between parent and child via the secondary analysis and integrative synthesis of two prior M-GTA studies, addressing a gap in the transitional care literature for conditions complicated by family roles and living-donor decisions.

Specifically, this study aimed to clarify the process of transitioning daily responsibilities between AYAs with biliary atresia who survive with their native livers and their parents. We conducted a secondary analysis of two prior qualitative studies [8,10], using newly applied analytical themes, and integrated the findings to construct a comprehensive model of the transition process.

In doing so, we have explored how AYAs and their parents experience and negotiate this shift in responsibilities, and how such a process can be understood as relational progress. This study contributes to more individualized and family-centered approaches to transitional care by elucidating these dynamics.

### 1.3. Summary of Prior Process Models

To provide a theoretical foundation for this study, we summarize two prior qualitative studies that explored the transition of daily responsibility in adolescents and young adults (AYAs) with biliary atresia who survive with their native livers—one from the AYAs’ perspective, focusing on their process of self-management, and the other from the parents’ perspective, focusing on how they gradually transfer responsibilities [8,10].

#### 1.3.1. Process of Daily Life Organization in Adolescents and Young Adults with Biliary Atresia Who Survive with Their Native Livers (Based on Hiratsuka [8])

Hiratsuka [8] described how AYAs with biliary atresia gradually organize their daily lives in response to their condition, presenting five core categories that reflect circular and developmental changes shaped by emotional and relational factors.

The process begins with “*The budding of a sense of ownership*”, where AYAs begin to recognize their illnesses and daily lives as their own. This awareness develops through tension among three elements: difficulty relating daily life to themselves, discomfort from social or physical cues, and withdrawal due to loss of control.

This is followed by “*Organization of daily life through the accumulation of experience*”, involving trial and error in building routines and developing self-management. Due to a few overt symptoms, AYAs may initially struggle but gradually learn to create personalized care strategies, including selective disclosure. “*Increased interest in living for the future for themselves and their parents*” reflects how AYAs, upon learning about medical risks or potential LDLT, start re-evaluating their lives. They strive for psychological balance and consider how to live meaningfully for both them and their parents.

Simultaneously, AYAs experience “*Confirmation of attachment to parents through the possibility of LDLT*”. The idea of receiving a liver from a parent intensifies their emotional conflict between independence and dependence. Over time, they emotionally re-anchor their bond with their parents and gain motivation to manage their health.

“*Understanding and consideration of people around them*” forms a contextual base throughout this process. Support from others helps AYAs move past resignation and fosters engagement with self-care. Thus, the process is non-linear and involves repeated oscillation between autonomy and vulnerability.

**Figure 1:**
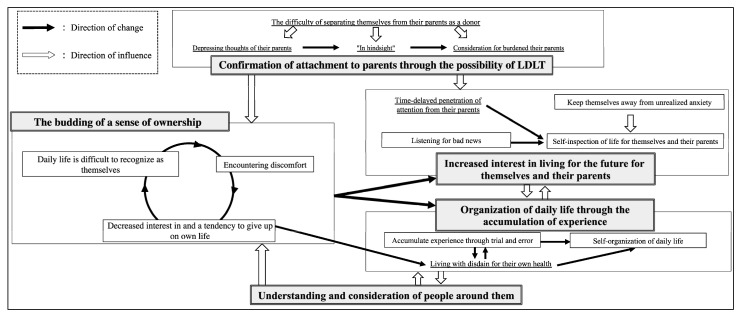
Process of daily life organization in adolescent and young adult patients with biliary atresia who survive with their native livers. Note: based on Hiratsuka [8].

This narrative is visually summarized in Figure 1.

#### 1.3.2. Process Through Which Parents Transfer Medical Treatment Responsibilities to Their Adolescent and Young Adult Children with Biliary Atresia Who Survive with Their Native Livers (Based on Hiratsuka et al. [10])

Hiratsuka et al. [10] described how parents gradually transfer responsibilities for medical treatment to their adolescent and young adult children with biliary atresia, presenting five core categories that reflect fluctuating emotional and relational adjustments over time.

The process begins with living a life of *cherishing the “now” of the child (CG1)*, shaped by the child’s life-threatening medical history and the desire to treasure the present. When faced with the demands of disease management, parents often *put the brakes on the child’s life (CG2)*, limiting their autonomy due to anxiety and fear.

As the child’s independence emerges, parents enter a phase of *learning as they go (CG3)* in which they balance monitoring the child’s health with the need to support self-management. This leads to tension between *CG2* and *leaving the child’s life to the child (CG4)*, which fluctuates based on health status and confidence.

An underlying issue is *conflict over their inability to be open about living-donor transplantation (CG5)*. Parents grapple with emotional ambivalence, which may reinforce *CG2* or, conversely, motivate *CG4* when autonomy becomes a priority for the child’s future.

These five interrelated categories illustrate a non-linear but progressive trajectory in which parents oscillate between control and release. The process is shaped by emotional state, the child’s health condition, and transplant-related decisions.

**Figure 2:**
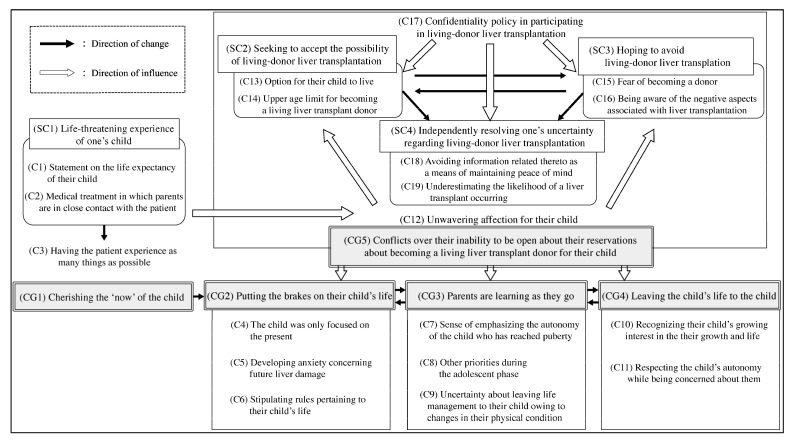
Process through which parents transfer medical treatment responsibilities to their adolescent and young adult children with biliary atresia who survive with their native livers. Note: based on Hiratsuka et al. [10]. CG, category; SC, subcategory; C, concept.

This narrative is visually summarized in Figure 2.

While all studies yielded practical, domain-specific process models, they offered independent and one-sided views. However, for AYAs with biliary atresia, the transition of responsibilities typically unfolds within the parent–child relationship. Therefore, a dyadic perspective is essential for understanding the intersubjective dynamics of shared responsibility and informing family-centered transitional care.

The present study builds upon these two earlier studies by reanalyzing their combined data from a relational viewpoint to develop a comprehensive, process-oriented model of responsibility transition.

## 2. Materials and Methods

### 2.1. Study Design

A qualitative descriptive design was employed, which is often used when knowledge about a topic is limited and rich, thus requiring contextualized explanations [12,13]. This study conducted a secondary analysis of interview data originally collected between November 2016 and August 2017 in two separate but methodologically aligned qualitative studies: one involving AYAs with biliary atresia [8] and the other focusing on their parents [10]. Both prior studies were conducted by the same research team using the modified grounded theory approach (M-GTA) [14,15] as the analytic method and involved matched AYA–parent dyads.

In the present study, we also adopted the M-GTA to reanalyze the full set of transcripts from a relational perspective within the qualitative descriptive design. No new data were collected.

The analysis comprised two phases: (1) a dyadic secondary analysis to clarify the trajectory of the responsibility transition between AYAs and parents; and (2) an integrative synthesis combining these findings with results from the two original studies to construct a comprehensive process model.

### 2.2. Population

This study focused on AYAs with biliary atresia who survive with their native livers and their parents as analytic dyads, with each AYA–parent pair treated as a unit of analysis.

AYAs were eligible if they were between the ages of 16 and 29, diagnosed with biliary atresia, had native livers, and possessed sufficient cognitive and emotional capacities to reflect on their life experiences. Parents were included if they were the primary caregivers actively involved in their child’s medical and daily care.

The exclusion criteria included being independent and living apart from their parents due to marriage or having established a new family, having an intellectual disability or a diagnosed mental disorder, undergoing major treatment, or lacking approval from the primary physician. Eligibility was assessed by the primary physician—typically a hepatologist or pediatrician—based on emotional stability, treatment status, and the ability to reflect on life experiences. While we anticipated the possibility that some individuals might be excluded due to emotional instability, sensitive treatment circumstances, or other concerns, no participants were excluded for emotional or clinical reasons.

Participants were recruited through nursing staff at affiliated hospitals. Written informed consent was secured from both AYAs and their parents using leaflets. We also obtained parental permission before approaching the AYAs, as they were minors.

A total of eight AYA–parent dyads participated in this study (Table 1). The ages of the AYAs ranged from 17 to 25. Seven were female, three were employed, and five were students. Two AYA participants (C and E) were living independently for educational purposes but had moved out of the family home after consulting with their parents post-high school graduation; they maintained frequent communication and continued to receive parental support for medical care, thereby fulfilling the relational criteria for inclusion. One participant (D) received a living-donor liver transplant at age 14; only her pre-transplant experiences were included in the analysis. Although she no longer met the criterion of being a native liver survivor, her case was included based on theoretical sampling. Her ability to reflect on adolescence with a native liver allowed for meaningful contrastive insights into the influence of transplantation on the transition process while remaining consistent with the study’s aim.

The parents included one father and seven mothers, all of whom were biological and primary caregivers involved in outpatient visits and treatment support.

Interview durations ranged from 26 to 73 min (mean: 44 min; SD: 14.7) and from 13 to 70 min (mean: 39 min; SD: 20.3) for AYAs and parents, respectively.

The sample size was determined based on the study’s narrow focus and the homogeneity of the participants. While there is no standard for adequate sample size in qualitative research [16], prior studies have demonstrated that small samples may be sufficient when objectives are clearly defined and populations are homogeneous [17].

### 2.3. Instruments

No new data or instruments were created for this secondary analysis. This study used the full original interview transcripts from two prior studies [8,10], integrating both datasets to examine parent–child dynamics from a relational perspective.

During the original data collection phase (2016–2017), semi-structured interview guides were developed separately for AYAs and parents to explore how families manage the transition of health-related responsibilities. In this secondary analysis, we referred to those original guides—created by the same research team—to interpret the transcripts. Below are illustrative items from those guides, presented to clarify the thematic focus of the interviews.

Key AYA questions included the following:

- “How do you and your parents share daily and health-related responsibilities?”
- “How has your role changed over time?”
- “What do you talk about with your parents regarding your illness or future, including liver transplantation?”

Key parent questions included the following:

- “How have you supported your child’s care and routines?”
- “When and how did you explain the diagnosis to your child?”
- “How do you discuss the future or transplantation?”

All interviews were conducted in Japanese (participants’ native language), delivered in a face-to-face format, and audio-recorded with consent. Interviews were conducted by the first author, a nurse experienced in AYA care and formally trained in graduate-level qualitative interviewing. Follow-up questions were flexibly adapted to individual responses, and all interviews were transcribed verbatim for analysis.

### 2.4. Data Collection

This study used data originally collected between November 2016 and August 2017 as part of two qualitative studies: one with AYAs with biliary atresia, conducted by Hiratsuka (2021) [8], and the other with their parents, conducted by Hiratsuka et al. (2021) [10]. The present study examined the same participants—eight AYA–parent dyads—in the secondary analysis to explore dyadic processes in the transition of care responsibility.

The two studies involved the same participant group—eight AYA–parent dyads—and individual semi-structured interviews were conducted with each member of the pairs. Semi-structured face-to-face interviews were conducted in the pediatric and transplant surgery outpatient departments of two advanced-care general hospitals located in an urban area of Japan.

Although the data were collected prior to the COVID-19 pandemic, their relevance to current clinical practice remains intact because the study focuses on the structural dynamics of relationships between AYAs and their parents in the context of chronic illness—phenomena that are relatively stable over time and less susceptible to short-term societal changes such as those caused by the pandemic. Moreover, the pre-pandemic nature of the data allows for a clearer understanding of foundational transition processes, free from the confounding effects of emergency health policies or shifts in care modalities like telemedicine.

### 2.5. Data Analysis

#### 2.5.1. Analysis in the Two Prior Studies

The two prior studies—Hiratsuka (2021) [8] on AYAs and Hiratsuka et al. (2021) [10] on parents—used the M-GTA to explore individual experiences of daily life and disease management. Although dyadic relationships were considered in the recruitment and interviews, the analyses focused on individual narratives without examining interdependent processes. The studies were conducted by the same research team using consistent methods and ethical protocols, and the participants were matched AYA–parent dyads, enabling their combined reanalysis in the present study.

#### 2.5.2. Secondary Analysis Using Modified Grounded Theory Approach

This study adopted the M-GTA, a qualitative approach developed by Dr. Yasuhito Kinoshita, to examine dyadic interactions in complex social contexts [14,15]. Unlike the original grounded theory approach (GTA) [18,19], the M-GTA prioritizes narrative continuity and participant perspectives, allowing researchers to explore embedded social meanings, making it especially suitable for reanalyzing the transition of care responsibilities between AYAs and their parents.

In this study, we reanalyzed the original verbatim transcripts from interviews with eight AYAs and their respective parents. While the original analyses [8,10] treated AYAs and parents separately, we approached each pair as a dyadic unit in the current analysis. By redefining the analytic theme as “the process for the transition of daily responsibilities from parents to AYAs with biliary atresia,” we refocused the analysis on their interactive relationship. This approach enabled us to explore how the AYAs and parents mutually influenced each other during the transition of care responsibility.

Following M-GTA procedures [15], narratives concerning the interactions between AYAs and their parents were extracted from the original verbatim interview transcripts collected in the prior studies. These narratives were selected based on analytic theme and focus and encompassed interactions related to parent–child relationships, emotional responses to illness and treatment, and treatment-related behaviors. Using an analysis worksheet, each concept was first defined based on a representative narrative example, followed by the assignment of a concept name and the addition of theoretical memos. A continuous comparative analysis was then conducted to identify similar examples that matched the defined concepts, and concepts were refined through the iterative addition of new data. In this secondary analysis, the concepts and categories identified in the two prior studies were referenced to complement interpretation, particularly in transitioning the analytic unit from individuals to dyads. Given the dyadic and abstract nature of the data and responsibility transition process, respectively, this study did not construct a narrative or hierarchical category structure typical of the M-GTA. Instead, the findings were organized into inductively derived phases.

All analyses were conducted manually to ensure direct engagement with the data in accordance with M-GTA principles. This approach reflects the methodological emphasis of the M-GTA on maintaining narrative continuity and interpretive sensitivity through close, reflective interaction with participant narratives, rather than relying on coding software.

Theoretical saturation was identified across phases, in line with the study’s aims and theoretical coherence, validating the adequacy of the final sample size [14]. Concept- and theory-level validities were qualitatively verified, and usability was discussed in terms of practical relevance in healthcare settings [14,15].

#### 2.5.3. The Integrative Process and Development of the Process Model

In addition to the dyadic secondary analysis, we conducted an integrative synthesis to develop a comprehensive process model describing the transition of daily responsibilities from parents to AYAs with biliary atresia who survive with their native livers. This synthesis incorporated findings from the current analysis and the two prior studies [8,10], which had independently examined AYA and parent perspectives. While these studies provided valuable insights into individual adaptations and role transitions, they did not explicitly address the relational dynamics between AYAs and their parents. To fill this gap, we reframed the analytic theme to focus on dyadic interaction processes and re-examined the dataset relationally.

Through this integrative process, we identified shared temporal structures and recurring phenomena across all three analyses. These elements were organized into a unified analytical framework reflecting the dynamic, non-linear nature of responsibility transfer within family systems. The resulting model captures how responsibility shifts bidirectionally in response to changing health status, evolving family roles, and major life events. It reflects the fluctuating, context-sensitive, and intersubjective characteristics of this transition and provides a practical foundation for care strategies that address both individual trajectories and relational dynamics in chronic illness.

To ensure the overall credibility and practical applicability of the findings, the analysis process was continuously supervised and reviewed by two pediatric nursing researchers with extensive experience in qualitative methods and adolescent care.

### 2.6. Ethical Considerations

This study is a secondary analysis of qualitative data collected in 2017, originally approved by the Ethical Review Committee of the Graduate School of Nursing, Chiba University, Japan (No. 28-58). The original study, which explored the life experiences of AYAs with biliary atresia and their parents, included a plan for analyses at three levels: AYAs, parents, and dyads. Written informed consent was obtained from all participants, including permission for the use of de-identified data in related secondary analyses.

This secondary analysis adhered to established ethical standards for reusing qualitative data [20]. All procedures remained within the scope of what the participants originally consented to. The dataset was de-identified, and reinterpretation was conducted for the original context and meaning. No new ethical issues arose, as the study remained aligned with the approved framework.

Although the focus on dyadic relational processes was not explicitly stated in the original protocol, it fell within the scope of secondary-use consent. Participant anonymity, dignity, and autonomy were maintained, and data were handled with ethical rigor and methodological transparency.

## 3. Results

### 3.1. Phases in the Transition of Care Responsibility

Four phases were identified in the transition of care responsibility based on a secondary analysis of previously collected qualitative data. These had not been explicitly structured as a continuous process in the two prior studies, which separately analyzed AYA and parent narratives, but emerged through the current dyadic and relational reanalysis. Following M-GTA principles, narrative integrity was prioritized, and category or narrative development was intentionally withheld. Instead, four inductively derived phases were identified to reflect relational shifts within each dyad. This structure was chosen to capture how key transitions arose not from incremental individual changes but from moments when AYAs and parents became attuned—or failed to become attuned—to each other’s evolving perceptions and behaviors.

While the prior studies focused on individual experiences, the present analysis highlights intersubjective turning points in mutual adaptation. Each phase is described below with illustrative dyadic excerpts from the interviews.

#### 3.1.1. Parent-Led Recuperation

“Parent-led recuperation” was a phase in which parents were responsible for most of the medical decisions, and AYAs were indifferent to the arrangement of their medical treatment, as though it were someone else’s concern. Some expressed doubts or negative feelings regarding treatment, even during adolescence and young adulthood. This was the first phase in the transition of daily responsibility, wherein parent-centered medical care was established through obedience to parents.

For example, Patient C did not question her parental supervision:

(Interviewer: Did you ever question or ask ‘Why?’ when they restricted your recuperation behavior or daily activities?)

Patient C (19 y/o, female):

I never did. I did as I was told.

(Interviewer: When did you start letting her manage her own medication and make decisions about exercising?)

Mother C (50 s):

Yes. When she was little, I did everything. I think it was when her medication started to decrease that I started to leave it to her. That was when she was in junior high school. Until then, I was in charge.

Conversely, there were cases wherein AYAs felt uncomfortable following their parents’ orders, and although the parents sensed their discomfort, “parent-led recuperation” continued. Patient E and her mother expressed this kind of treatment management in their respective narratives:

Patient E (21 y/o, female):

I thought the clothes were cute, but I didn’t like it when they told me to wear tights.

Mother E (40 s):

Even if I explain to her about the disease, she doesn’t understand, so she is, for lack of a better word, under our control. Parents can freely control their children, and if they say no, it’s no good.

At this stage, AYAs had not yet developed or asserted their own competence in managing their condition. Their limited involvement in medical decisions may reflect both a lack of perceived capacity and the strength of established parent-led routines. As such, the potential for patient-centered care remained latent, emerging more clearly in subsequent phases.

This phase was observed across multiple dyads, including C, D, and E, with varying forms of interaction regarding how AYAs responded to parental control. While excerpts from Dyads C and E are representative examples, similar patterns of parent-led care and limited AYA involvement were evident in several dyads. Although the intensity and context of parental control varied, the commonality across these dyads indicates the presence of a recurring interactional pattern during this phase.

#### 3.1.2. Vicious Cycle of Control and Rebellion

“Vicious cycle of control and rebellion” was the phase wherein AYAs’ autonomy increased as parent-centered medical treatment continued, leading to heightened tension between AYAs and their parents regarding who should bear the weight of responsibility. This increase in tension was often preceded by a change in the AYAs’ perceptions and actions. First, AYAs felt uncomfortable with their illnesses and how they had allowed their parents to manage their lives; they began to intentionally violate parental restrictions or stopped adapting their lives to their illnesses. Conversely, as the youth reached adolescence and became more independent, their parents became confused and were unable to support their attempts to adjust their recuperative lifestyle. Parents felt that they had to fulfill their responsibilities, which further emphasized their caution. The AYAs sensed their parents’ disapproval of them and became more rebellious, continuing to act out of desperation.

Patient E, who felt uncomfortable leaving the management to her parents during the “parent-led recuperation” phase, progressed to a “vicious cycle of control and rebellion” due to her increased desire for freedom and rebelliousness toward her parents:

Patient E (21 y/o, female):

I was really rebellious against my parents! … When we went shopping for clothes, my sister could choose what she liked, but I had to choose for warmth. I was like, ‘Why?’ I felt like my mother was really obsessed with my physical condition.

In response to Patient E’s change, Mother E, while she understood her daughter’s discomfort and rebellion, felt that she had to increase her attention and tried to enforce tighter restrictions on her daily life:

Mother E (40 s):

As my daughter entered middle school, she began to ask herself, ‘Why can’t I do what everyone else can?’ She began to be reckless. For example, she started staying up late at night. So, we made a curfew in our house. But she didn’t like the rule itself… She didn’t agree.

Patient B believed that she was living life on her own terms. However, the feeling that her parents disapproved of this intensified her rebelliousness. Patient B’s thoughts were supported by her father’s statements:

Patient B (18 y/o, female):

I kind of pushed myself too hard while playing. My parents told me that I was catching colds because my body was tired. They said my immune system was weak or something. I thought that was too much… I knew my body was tired, so I tried to adjust by going to bed early and so on. I was taking care of myself in my own way, so I didn’t want my parents to nag me about it.

Father B (60 s):

I also told my daughter not to do anything too exhausting, I think she wanted to rebel against us as parents. She pushed herself to continue activities in the brass band without listening to me.

In this phase, AYAs began to develop awareness of their condition and asserted their own judgment about how to live with it, even if it conflicted with parental expectations. Their efforts to take control of their recuperation sometimes clashed with parental caution, revealing the difficulty of renegotiating responsibility during the transition period.

Events such as a referral to a transplant surgeon or a change in the adolescent or young adult’s physical condition further heightened parental attention and anxiety, reinforcing the dynamic tension between growing AYA independence and parental oversight:

Mother A (50 s):

When my daughter was in eighth grade, the transplant surgeons became involved, and if she was even a little bit sick, I was like, ‘Oh, what if she gets cholangitis again?’… (To her daughter), ‘Oh, can’t you care a little more?’

This phase was observed in multiple dyads, such as A, B, and E, with variation in how mutual frustration and conflict played out. Across these cases, common elements included increased AYA resistance, intensifying parental vigilance, and emerging cycles where both parties’ responses reinforced the other’s distress. The recurrence of these interactional patterns across dyads suggests a distinct phase in the transition of care responsibility, representing a period of relational tension that challenged mutual trust and communication.

#### 3.1.3. Passing the Axis of Responsibility

“Passing the axis of responsibility” refers to the beginning of patient-centered medical treatment, which started when the responsibility shifted from parents to AYAs. This phase mainly unfolded in a step-by-step and planned manner. However, “passing the axis of responsibility” was established only when the AYAs changed their perceptions and actions regarding taking responsibility for their lives in response to their parents’ behaviors, which then resulted in a change for both the AYAs and their parents.

Mother E gradually handed over responsibility to Patient E in anticipation of the future, wherein she would eventually live alone:

Mother E (40 s):

She’s always wanted to leave the house… so I rented a spare room at her grandma’s house for her to practice and get some experience of living on her own.

Mother C fulfilled Patient C’s wish to live alone when she reached college age. Patient C described how she changed her behavior after her mother’s actions:

Mother C (50 s):

She always said she wanted to live on her own, so I left it up to her. I did at least warn her not to go to bed too late, but I tried not to pay too much attention.

Patient C (19 y/o, female):

Living alone is tough in its own way, but I think I am able to take care of my eating habits. Since my parents don’t live with me, I feel a sense of responsibility that I have to manage everything myself.

Conversely, there were cases wherein the phase of “passing the axis of responsibility” occurred in conjunction with time-related events, such as transitioning to higher education or employment. The transition of daily responsibility occurred even when the AYAs had not undergone the necessary changes in perception and actions for the transition, or when the parents did not realize in advance that their children were not ready to accept and fulfill their responsibilities. Consequently, the AYAs’ health conditions worsened, and parents were forced to resume responsibility for them, reverting to the phase of “vicious cycle of control and rebellion.” This forced regression analysis was expressed in the narratives of Patient H, who experienced physical changes after leaving his parents and entering the workforce:

Patient H (25 y/o, male):

The gas station where I worked before was a pretty physically demanding job. When I was working there, there were periods when the results of my blood tests were poor, and I wondered what I could do. It was beyond my ability to adjust and manage my own life. I had not told the company about my illness. I wanted to work, keeping my illness hidden.

Mother H (50 s):

When I advise my son about his daily life, he often says, ‘I know!’ And I say, ‘If you knew what you were doing, you wouldn’t have done this!’ After my son entered the workforce, he and I continued to have this exchange. He never told his boss about his illness. Without my knowledge, he worked just like a normal,… healthy young man. When the doctor saw the results of his blood test, he said, ‘It’s pretty bad.’ At the time, he was renting an apartment, and we only had occasional contact with him. I panicked and forced my son to move back home immediately.

This phase marked the emergence of patient-centered recuperation, wherein AYAs began to demonstrate judgment, responsibility, and independence in their self-care. The handover of responsibility was not unidirectional; rather, it involved dynamic mutual adjustments between AYAs and their parents, shaped by changes in physical condition, living circumstances, and relational expectations. The transition was not always stable—when the transfer occurred prematurely or without sufficient relational support, regression to earlier phases was observed.

This phase was observed across multiple dyads, such as A, B, and E. The identification of this phase emerged through recurrent narrative patterns that highlighted the difficulty and fragility of sustaining independent care without mutual readiness. These features were consistently observed in the dyadic data, conceptually saturating this phase as an interactional turning point in the process of transitioning care responsibility.

#### 3.1.4. Aligning the Parent–Child Rhythm to Create a Patient-Centered Life

“Aligning the parent–child rhythm to create a patient-centered life” was the final phase of transitioning daily responsibility from parents to AYAs. The relationship between the AYAs and their parents, wherein the parents advise the AYAs about their lives, continued; however, it was no longer unidirectional from parents to AYAs, as it had been in the previous phases.

The AYAs proactively organized their daily lives in cooperation with their parents. This required them to adopt a shared perspective that incorporated the AYAs’ opinions and physical experiences into daily management. Even if the AYAs’ conditions worsened, neither they nor their parents reacted with the anxiety observed in the previous phases. Instead, they could discuss and negotiate the appropriate next steps.

The characteristics of this phase are expressed in the narrative of Patient G, who experienced acute cholangitis:

Patient G (24 y/o, female):

My doctor said, ‘In actuality, we don’t know the cause (of the sudden onset of cholangitis).’ So, my mother and I both said to each other, ‘If my doctor doesn’t know, there’s nothing we can do about it.’ My mother and I are this way.

In the event that the attending physician recommended that the AYAs visit a hospital that performs organ transplants, the perceptions of the AYAs and their parents regarding the possibility of LDLT came to the forefront. Even if the topic of living-donor liver transplantation was not completely open for discussion between the AYAs and their parents, they began to prepare for the possibility.

When a doctor presented the possibility of a living-donor liver transplant, Mother E began discussing her daughter’s future with her:

Mother E (40 s):

My daughter told me that the doctor talked to her about a transplant. She said she didn’t want a living-donor liver transplant… She said, ‘I want to live with my native liver if possible.’ We then talked about what we would do to make that happen.

In this phase, most AYAs were aware of the possibility of living-donor liver transplantation, as was the case with Patient E. Unlike in the previous three phases, where such awareness was often vague or unshared, the AYAs and their parents in this phase acknowledged and understood each other’s perspectives regarding the transplant. This phase represented a mature stage of patient-centered recuperation, where AYAs not only took responsibility for their care but also engaged in shared decision-making with their parents. The rhythm of care was collaboratively adjusted, and trust was evident even in the face of medical uncertainty.

This phase was identified across dyads, such as G and E, and conceptually saturated through repeated narrative patterns that showed stable mutual adjustment, communicative alignment, and shared awareness of long-term medical contingencies, such as transplantation.

### 3.2. Supplementary Analysis Integrating Prior Studies for Process Model Development

To develop the “Process Model for Transitioning Care Responsibility to Adolescents and Young Adults with Biliary Atresia,” we conducted a supplementary analysis that integrated findings from the two prior studies [5,7] with the results of the current dyadic analysis. In 2021, Hiratsuka [5] focused exclusively on AYA narratives, describing their individual processes of adapting to daily life with biliary atresia. In the same year, Hiratsuka et al. [7] explored the gradual transfer of medical responsibility from the perspective of parents. While both studies yielded valuable insights, neither captured the dynamic, reciprocal nature of transitioning responsibilities within parent–AYA dyads.

**Figure 3:**
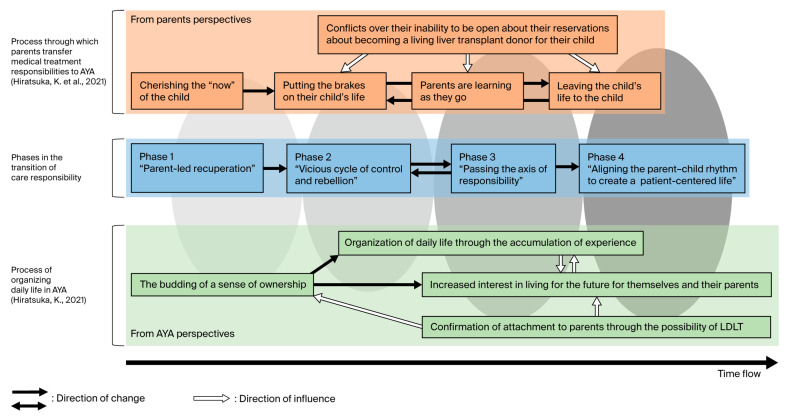
Process model for the transition of daily responsibility from parents to adolescents and young adults with biliary atresia. Joint display of the process model and prior findings. Ovals indicate the relationship between the process phases and the key categories derived from the two prior studies, functioning as a joint display that integrates the perspectives of AYAs and parents. Note: This figure illustrates the transition of daily responsibilities from parents to AYAs with biliary atresia who survive with their native livers. The model was developed through a secondary and integrative analysis of two prior studies [8,10]. The horizontal axis in the center (blue area) represents four inductively derived phases: “parent-led recuperation,” “vicious cycle of control and rebellion,” “passing the axis of responsibility,” and “aligning the parent–child rhythm to create a patient-centered life.” Arrows indicate directions of change and influence over time. Ovals represent key conceptual categories identified in the prior AYA-focused [8] and parent-focused [10] studies. These categories are visually mapped onto the four process phases to show how the original findings informed the current model. Thus, the ovals function as part of a joint display, integrating insights from both primary and secondary analyses and demonstrating how perspectives from AYAs and parents interweave and evolve throughout the transition process.

The present analysis reconceptualized the transition of responsibility as an interactive process of mutual behavioral adjustment. Using the current findings as an axis, we revisited the categories and subcategories from the two prior studies [5,7], aligning them along shared events and temporal markers. This integrative approach enabled the construction of a unified process model (Figure 3) that depicts the evolving interaction between parents and AYAs.

The model illustrates how the transition of responsibility begins with changes in perception or behavior in either the parent or the adolescent or young adult, which then prompts a response in the other party. These mutual adjustments ultimately shape the trajectory of the dyadic relationship. This dynamic interaction aligns with symbolic interactionism, the theoretical foundation for our analysis, which posits that meaning is created through social interaction and that observable behaviors, including deliberate non-responsiveness, are acts that carry significance within relationships.

Although the model does not impose a rigid chronological sequence, a timeline was incorporated to support clinical usability. This visualization allows healthcare professionals to better identify gaps in mutual readiness—particularly in relation to perceptions of potential living-donor liver transplantation—and to time interventions accordingly.

The relationships between the four identified phases and categories from the two prior studies are summarized as follows:

- In the “parent-led recuperation” phase, AYAs demonstrated [a budding sense of ownership], while parents exhibited [a cherishing of the “now” of the child] and [putting the brakes on their child’s life]. Emerging discomfort in AYAs signaled early tension within the dyad.
- In the “vicious cycle of control and rebellion” phase, conflict escalated, with the persistence of [a budding sense of ownership] among AYAs and [parents learning as they go]. Parental overinvolvement often intensified during episodes of worsening health or physician referrals.
- The “passing the axis of responsibility” phase was associated with AYAs’ [organization of daily life through the accumulation of experience] and parents’ [decision to leave the child’s life to the child]. However, misalignment in readiness sometimes caused health deterioration and regression to earlier phases.
- The “aligning the parent–child rhythm to create a patient-centered life” phase reflected increasing alignment in perceptions of living-donor liver transplantation. AYAs engaged with [confirmation of attachment to parents through the possibility of living-donor liver transplantation], while parental [conflicts over their inability to be open about their reservations] diminished. These shifts facilitated a collaborative life structure.

In the final integrated phases, complete independence was rarely observed. While AYAs with other chronic conditions may achieve full autonomy as part of their developmental process, those with biliary atresia who survive with their native livers tend to maintain psychological and relational closeness with their parents. This appears to be influenced by the ongoing possibility of requiring living-donor liver transplantation.

In the latter two phases of the identified process model—“passing the axis of responsibility” and “aligning the parent–child rhythm to create a patient-centered life”—patient-centered competence was observed to develop gradually over time. AYAs were seen organizing their daily routines, making decisions based on their physical condition, and engaging in shared discussions with their parents about potential future events, including liver transplantation. These activities included meal planning, medication management, scheduling rest, and planning for the future. Such behaviors were shaped through ongoing interactions with parents rather than emerging in isolation.

The model shows that AYAs with biliary atresia progressively demonstrated patient-centered actions and decision-making while remaining within sustained family relationships. The final phases were characterized by a form of interdependent autonomy in which responsibility was balanced through mutual adjustment rather than through unilateral independence. This pattern of care responsibility transition was consistently observed in several dyads during the final phases and contributed to the development of the current process model.

## 4. Discussion

### 4.1. The Influence of Individual and Contextual Factors on the Transition Process

In this section, we examine how various individual and contextual factors influenced the progression of the four phases—identified through secondary analysis—that comprise the trajectory of transitioning responsibilities between AYAs and their parents with biliary atresia who survive with their native livers.

Developmental age and maturity were generally associated with progression across phases, particularly from adolescence to young adulthood. This finding aligns with previous studies on adolescents with chronic illnesses, which suggest that the gradual transfer of everyday health-related responsibilities from parents to AYAs typically follows developmental progression [21,22]. However, this was not always the case. For instance, despite being older, Patient H continued to demonstrate a pattern of control and rebellion, suggesting that chronological age alone is insufficient. Instead, autonomy—especially the ability to organize daily routines and make decisions—was more important. Notably, such autonomy did not develop in isolation but was cultivated through ongoing interactions with parents. The final phase, “aligning the parent–child rhythm to create a patient-centered life,” exemplified how autonomy emerged as a relational construct, particularly in the context of shared decision-making around future possibilities such as living-donor liver transplantation. This underscores that meaningful patient-centered competence is supported by mutual recognition and sustained support within the family unit.

Living independently or being employed also encouraged autonomy but did not directly determine progression. Instead, these factors acted indirectly by increasing self-management needs. Similarly, due to the gender imbalance in our sample (one male and seven female participants), it was not feasible to conduct a robust gender-based analysis. Nevertheless, female AYAs who communicated openly with their mothers tended to reach the final phase more consistently. This observation supports prior findings that mother–daughter dyads may experience more cooperative transitions than mother–son pairs [23]. This suggests that maternal involvement and gender-concordant parent–child pairs may help facilitate the transition. However, our findings suggest that the quality of the parent–child interaction appeared more decisive than gender alone.

The clinical course of biliary atresia also influenced the transition trajectory. Even during periods of stable liver function, adolescence and young adulthood introduced new physical and psychosocial stressors—such as pubertal growth spurts, educational changes, and employment-related pressures—that could trigger disease deterioration [24]. As shown in our prior study [8], fluctuations in health status heightened disease awareness for some participants, while for others, instability led to increased anxiety. In this secondary analysis, such episodes were found to either promote progression beyond the “parent-led recuperation” phase or, conversely, cause regression to earlier relational dynamics such as the “vicious cycle of control and rebellion.” These outcomes show that it is not disease or development alone but how AYAs and their parents navigate them together that shapes the transition—sometimes advancing it, sometimes setting it back.

Lastly, although variables such as parental education, occupation, and social resources were not examined in this study, they may influence the transition indirectly by shaping parenting and family interactions. Future research should explore these broader influences.

While “passing the axis of responsibility” appears promising for structured support, our findings suggest that effective nursing interventions may be applicable across all four phases, depending on each family’s situation. Clinically, understanding age, developmental maturity, and contextual variation is key to interpreting the trajectory of transitioning responsibilities and determining when and how to intervene appropriately.

### 4.2. Evaluation of the Usefulness of the Developed Process Model

The developed process model captures the transition of daily responsibility between AYAs with biliary atresia who survive with their native livers and their parents, focusing on how their perceptions and actions evolve. It also highlights shifting perceptions of living-donor liver transplantation (LDLT) over time and the differing awareness of this option between AYAs and parents.

The two prior studies examined AYAs and parents separately [8,10], despite the recognized importance of interactions between them—particularly given that parents may become living donors. Due to the emotional and ethical complexity of LDLT [25], healthcare providers have rarely implemented specific interventions to support AYA independence during this transition.

Badour et al. [26] noted that parents should adapt their involvement as their children grow. Parental involvement and appropriate changes in the parent–child relationship are crucial for promoting independence in AYAs with chronic illness. Our model offers clinicians a framework to assess family readiness, guide role transitions, and co-develop individualized care plans.

Previous reviews have also highlighted the negative impact of excessive parental control [26]. Without sufficient preparation, families may fall into a “vicious cycle of control and rebellion” or fail to pass the axis of responsibility. By highlighting reciprocal interactions, this model illustrates how shifts in perceptions and behaviors affect the transition process and helps professionals recognize stagnation and respond appropriately.

For example, in the “parent-led recuperation” phase, nurses can recognize early expressions of doubt or hesitation by AYAs and facilitate shared reflection between parents and children. During the “vicious cycle of control and rebellion” phase, clinicians can intervene to de-escalate conflict and support mutual understanding by interpreting underlying emotional dynamics. In the “passing the axis of responsibility” phase, nurses can foster early readiness for responsibility by facilitating structured communication and supporting shared decision-making. Finally, in the “aligning the parent–child rhythm” phase, they can help co-create individualized care plans that honor both AYA autonomy and the continued role of family support.

By making these phase-specific interventions visible, the model offers clinicians a developmentally grounded and relationally attuned framework for supporting AYAs during the transition from parent-led to patient-centered care. It provides actionable guidance for tailoring nursing support to the shifting dynamics of each family, ultimately enhancing readiness for independent disease management while preserving meaningful familial relationships.

### 4.3. Limitations

This study has several limitations regarding generalizability. The small sample size, limited data sources, and lack of observational data or simultaneous interviews with AYAs and parents restrict the depth and breadth of the findings. Consequently, inconsistencies or ambiguities in individual narratives cannot be fully ruled out.

The participants were limited to AYAs with biliary atresia who survive with their native livers. While this specificity enabled in-depth analysis, it narrows the applicability of the findings to populations with different disease profiles. Nonetheless, the developed model provides a valuable framework for understanding the unique experiences of this specific population while also offering conceptual insights that may inform the study of psychosocial transitions in other chronic illness contexts.

Although the original data were collected in 2016–2017, the psychosocial themes explored—such as autonomy, dependency, and evolving family dynamics—remain highly relevant. Still, we acknowledge that updated data would improve the timeliness and external validity of the findings.

To address these limitations, future research should pursue two key directions. First, validation studies with larger and more diverse samples of AYAs with various chronic conditions are needed to enhance the academic rigor and generalizability. Second, from a clinical perspective, practice guidelines should be developed based on this model, incorporating family dynamics, developmental stages, and disease-specific considerations. Advancing both these directions—academic validation and clinical application—will enhance the model’s utility in supporting AYAs during the transition to adult care.

## 5. Conclusions

This study developed a four-phase process model describing how AYAs with biliary atresia who survive with their native livers and their parents navigate the transfer of daily disease management responsibilities. The model—comprising “parent-led recuperation,” “vicious cycle of control and rebellion,” “passing the axis of responsibility,” and “aligning the parent–child rhythm to create a patient-centered life”—was constructed through the secondary analysis and integrative synthesis of two prior studies.

The model highlights the mutual adaptation between AYAs and parents through communication and negotiation, identifying relational turning points and cognitive–behavioral shifts that guide phase-specific nursing interventions.

Although one participant had undergone transplantation, their narrative focused on the pre-transplant period. Thus, the findings primarily reflect experiences of AYAs with native livers. While grounded in this context, the model’s relational dynamics may extend to other chronic illness populations.

Despite the time that had elapsed since the data collection, the enduring relevance of these psychosocial processes supports the model’s conceptual and clinical value. Future studies should validate and adapt it across broader settings to enhance transitional care practices. Clinically, individualized care strategies that account for family dynamics, developmental stages, and disease specificity are essential to supporting AYAs and their families in achieving sustainable, shared illness management.

## Figures and Tables

**Table 1:** Participant characteristics.

Participant	Patients’ Age (Years)/Sex	Patients’ Social Status	Patients’ Transplant Status	Parents’ Age (Years)/Relationship
A/a	17/female	High school/living with parents	Surviving with native liver	50 s/mother
B/b	18/female	University/living with parents	Surviving with native liver	60 s/father
C/c	19/female	University/living alone	Surviving with native liver	50 s/mother
D/d	19/female	Vocational college /living with parents	Surviving with living-donor liver transplant (age 14 at transplant)	40 s/mother
E/e	21/female	Working/living alone	Surviving with native liver	40 s/mother
F/f	24/female	University (online program)/living with parents	Surviving with native liver, awaiting LDLT	40 s/mother
G/g	24/female	Working/living with parents	Surviving with native liver	40 s/mother
H/h	25/male	Working/living with parents	Surviving with native liver	50 s/mother

## Data Availability

Data supporting the findings of this study are available upon request from the corresponding author. The data are not publicly available because of privacy and ethical restrictions.

## Public Involvement Statement

No public involvement in any aspect of this research.

## Guidelines and Standards Statement

This manuscript was drafted against the Standards for Reporting Qualitative Research (SRQR).

## Abbreviations

The following abbreviations are used in this manuscript:

AYAs	Adolescents and young adults
LDLT	Living-donor liver transplantation
M-GTA	Modified grounded theory approach
